# Use of a Novel Tele-Assessment Tool for the Identification of Autism in Preschool-Aged Children

**DOI:** 10.1007/s10803-025-07144-9

**Published:** 2025-12-06

**Authors:** Laura L. Corona, Amy Weitlauf, Liliana Wagner, Alexa Dixon, Tori E. Foster, Ambar Muñoz Lavanderos, Makayla Honaker, Amy Nicholson, Caitlin Stone, Amy Swanson, Alison Vehorn, Zachary Warren

**Affiliations:** 1 Vanderbilt Kennedy Center/Treatment and Research Institute for Autism Spectrum Disorders, Vanderbilt University Medical Center, Nashville, TN 37203, USA; 2 Department of Pediatrics, Vanderbilt University Medical Center, Nashville, TN 37203, USA; 3 Waisman Center, University of Wisconsin Madison, Madison, WI, USA; 4 Department of Psychiatry & Behavioral Sciences, Vanderbilt University Medical Center, Nashville, TN, USA; 5 Department of Special Education, Vanderbilt University, Nashville, TN, USA

**Keywords:** Autism, Tele-assessment, Preschool-aged

## Abstract

**Purpose:**

This work evaluates use of the TELE-ASD-PEDS-Preschool (TAP-P), a telemedicine-based autism assessment tool for the preschool age range. The TAP-P is a play-based instrument with varied administration and scoring procedures based on a child’s language level. This study compared tele-assessment using the TAP-P to in-person autism assessment.

**Methods:**

Participants were 116 children (aged 36–71 months) referred for autism evaluation. Participants first completed in-home tele-assessment (administration of the TAP-P, clinical interview, and Developmental Profile 4). All participants then completed in-person assessment with a different psychological provider, including cognitive or developmental assessment, adaptive behavior assessment, and autism-focused assessment (ADOS-2). Caregivers completed questionnaires after each appointment assessing their perceptions.

**Results:**

When asked to make a binary decision about presence or absence of autism, tele-assessment and in-person clinicians agreed for 82% of participants (n = 95). In most instances of diagnostic disagreement (n = 17), tele-assessment clinicians indicated the absence of autism and in-person clinicians indicated the presence of autism. When given the option to select “unsure,” tele-assessment clinicians reported uncertainty for 28% of participants assessed using Form 1 of the TAP-P (designed for children with less verbal language, defined as two-word phrases or less) and 52% of children assessed using Form 2 (for children with more verbal language). Families reported broad satisfaction with tele-assessment procedures.

**Conclusion:**

This work highlights potential utility of tele-assessment for identification of autism in preschool-aged children with less verbal language, while emphasizing the critical need for additional research related to use of tele-assessment for children using more complex and flexible verbal language.

Early autism identification through timely diagnostic evaluation is a consistent priority within pediatric care communities (IACC, 2023; Hyman et al., 2020). However, in spite of advancements in early screening, national data indicates that the median age of first time diagnosis remains above 4 years (Shaw et al., 2025). Wait times for diagnostic evaluation increase with age, compounding the “wait list problem” such that preschool-aged children frequently wait a year or more for evaluation (Brinster et al., 2023; Gordon-Lipkin et al., 2016; Kanne & Bishop, 2021). The persistent inability of diagnostic systems to provide prompt evaluations for young children has led to calls for adapting and creating models of care, including designing novel assessment tools and imagining a range of pathways through which a child may receive autism evaluation (Zwaigenbaum et al., 2021). In particular, telemedicine-based assessment (hereafter, tele-assessment) has presented options for addressing access barriers for some families (McNally Keehn et al., 2023; Shaw et al., 2025; Stainbrook et al., 2019; Stroupková et al., 2024; Wagner et al., 2020, 2022). However, few tools to date have focused specifically on preschool-aged children.

Tele-assessment of autism in young children gained popularity during the COVID-19 pandemic, with several tools adapted or developed during that period (Alfuraydan et al., 2020; Berger et al., 2021; Dahiya et al., 2021; Stavropoulos et al., 2022a, 2022b). Among these, the TELE-ASD-PEDS (TAP; Corona et al., 2020) has a strong research base, including broad uptake and use during the pandemic (McNally Keehn et al., 2023; Wagner et al., 2020, 2022), completion of a clinical trial comparing use of the TAP to in-person assessment (Corona et al., 2024), and an ongoing multi-site study (NCT05047224) establishing use and psychometric properties among a more diverse population (Gangi et al., 2025). Across published TAP tele-assessment work, diagnosis of autism can be confirmed or ruled out for many toddlers (Corona et al., 2024; Gangi et al., 2025; McNally Keehn et al., 2023; Wagner et al., 2020, 2022). This work has also highlighted clinician and caregiver satisfaction with tele-assessment procedures, measured using both percent agreement with items on satisfaction questionnaires (Corona et al., 2024; Gangi et al., 2025) and qualitative analysis of open-ended survey responses (Corona et al., 2020; Wagner et al., 2020).

Less research to date has focused on the use of tele-assessment with children in the preschool age range (i.e., approximately 3 to 5 years of age). Review of the literature identified three tele-assessment models for preschool-aged children. The first is the Brief Observation of Symptoms of Autism (BOSA; Lord et al., 2020), which shows preliminary promise (Dow et al., 2022; Stavropoulos et al., 2022a, 2022b) but relies on use of a standardized set of materials, limiting its scalability and use in many community settings. The second focuses on use of the TAP with older children, including pandemic use with children with minimal verbal language through 78 months of age (McNally Keehn et al., 2023) and a proposed model for use of the TAP with children through five years of age (Mirzaei et al., 2025). The third describes pandemic use of the “TELE-ASD-KIDS,” which incorporated activities from both the TAP and the ADOS-2 for children using spoken phrases or sentences (Stavropoulous et al., 2022b). Published research on use of the TAP with older children focused on its use during the pandemic, which precluded comparison to in-person assessment. However, existing work suggests that tele-assessment can be more challenging with older children, as language skills, play schema, and social complexity increase (Wagner et al., 2023).

This preliminary work on preschool tele-assessment emphasizes the need for tools designed specifically for this population. Given frequent extended wait times for evaluation (Brinster et al., 2023; Gordon-Lipkin et al., 2016), even children with clear early concerns may not receive diagnostic assessment before three years of age. Further, autism characteristics may not become apparent to families or providers until later in childhood for a variety of reasons, including phenotypic variability (Ozonoff et al., 2018), cultural variability in child development norms (De Leeuw et al., 2020), and lack of access to trained providers (Drahota et al., 2020; Durkin, 2015). As such, the application of tools developed for toddlers to this older age group may miss many autistic preschoolers.

The TELE-ASD-PEDS-Preschool (TAP-P) was developed to address this gap. The TAP-P was designed explicitly for the preschool-age range using a co-design process that involved caregivers, clinicians, and early intervention providers. Instrument development was informed by analysis of assessment data from 913 children between 37 and 72 months of age (see Wagner et al., 2023 for detailed description of the development process). The TAP-P is an interactive tele-assessment tool, in which clinicians guide children and their caregivers through a series of play and interaction activities via telehealth. Activities were designed to highlight a child’s communication skills, social interactions, and play behaviors, using separate sets of activities for children with less verbal language (i.e., operationalized as use of two-word phrases or less) and those with more verbal language (i.e., operationalized as flexible of use of phrases/sentences of three or more words).

In a pilot sample of 30 children who completed the TAP-P (Wagner et al., 2023), tele-assessment clinicians’ ability to accurately identify or rule out autism was higher for children with less verbal language (77% agreement with existing diagnosis) than for children using more verbal language (64% agreement with existing diagnosis). Clinician accuracy and confidence were also substantially higher when confirming existing autism diagnoses (80%) than when ruling out autism (30%). Clinicians broadly highlighted the need and value of a clinical interview with parents, rather than use of the TAP-P in isolation.

The present study (NCT05373173) extends this preliminary work on the TAP-P, focusing on use of the TAP-P within a tele-assessment protocol inclusive of clinical interview and developmental assessment. In this study, participating children received tele-assessment using the TAP-P, followed by traditional in-person assessment. This study focused on examining (1) diagnostic ascertainment via tele-assessment, (2) diagnostic concordance between tele-assessment and in-person assessment, and (3) caregiver and clinician perceptions of tele-assessment procedures.

## Method

### Participants

Eligible participants were children between 36 and 72 months of age referred for evaluation for possible autism by primary care providers, early intervention providers, or caregivers. Information about the study was provided to primary care offices and early intervention teams with established partnerships with our academic medical center. Study information was also posted in our institutional newsletters and websites designed to match potential participants to ongoing research. Families were invited to enroll in the study following provider or self-referral. Additional eligibility criteria included participation of a primary caregiver with (1) sufficient facility with English to complete study procedures and measures and (2) access to a device capable of supporting the video conferencing platform Zoom (e.g., phone with camera, tablet, laptop). Exclusion criteria included significant sensorimotor impairments that would interfere with participation in assessment activities.

In total, 116 participants completed both tele- and in-person assessment (n = 2 lost to follow-up, completed only tele-assessment; See Fig. 1 for study flow). Participants (28% female, 72% male) ranged from 36 to 71 months of age (m = 47.8 months, SD = 9.90), and 42% identified as being from a minoritized racial or ethnic group (See Table 1). Forty-seven percent of caregivers reported a household income below the state median income.

### Tele-Assessment Measures

#### TELE-ASD-PEDS-Preschool (TAP-P)

The TAP-P is a tele-assessment tool designed explicitly for evaluation of autism-related behaviors in preschool-aged children via telehealth. All children complete an initial set of clinician-guided, caregiver-mediated activities, including independent play, interactive play with caregiver, turn-taking, and bids for social responding. The TAP-P then has distinct sets of activities for use based on a child’s current expressive language level. TAP-P Form 1 is intended for children with less verbal language, operationalized as use of spoken language consisting of two-word phrases or less. Specifically, this may include children using no words, single words only (including learned units, e.g., “all done,” “thank you”), or two-word phrases. Form 1 includes additional caregiver-led activities focused on reciprocal play routines and opportunities to observe a child’s communication strategies. Form 2 is intended for children who regularly communicate verbally using flexible phrases or sentences of three or more words. As such, Form 2 includes activities designed to prompt for reciprocal conversation as well as interactive play. Form 2 includes activities designed for the child and caregiver, as well as opportunities for the child to interact directly with the assessing clinician. See Table 2 for description of activities.

The TAP-P is intended to be scored immediately after administration. Distinct scoring procedures are provided for Forms 1 and 2, with behaviors scored reflecting those behaviors identified during the tool development process that best distinguished children with autism from children without autism (Wagner et al., 2023). Scoring procedures use Likert scoring conventions of: 3 = behaviors characteristic of autism clearly present; 2 = possible behaviors consistent with autism, and 1 = behaviors characteristic of autism not present. Form 1 scoring, for children with less verbal language, requires that clinicians provide ratings on 10 behavioral anchors (see Table 3). Total scores can range from 10 to 30, with higher scores indicating increased presence of autism-related behaviors. Form 2 scoring, for children regularly using flexible phrases or sentences, includes ratings for 12 distinct behaviors (see Table 3). Total scores for Form 2 range from 12 to 36, with higher scores indicating increased presence of autism-related behaviors.

#### Developmental Profile, 4th Edition (DP-4)

The DP-4 (Alpern, 2020) is a clinician-administered caregiver interview focused on developmental skills in five areas: Physical, Adaptive Behavior, Social-Emotional, Cognitive, and Communication. Each item is scored as Yes/No regarding whether the child has attained the given skill. Norms-based standard scores are provided for birth through 21 years, 11 months for each domain, as well as a General Development Score. In past work, scores from administration of the DP-4 via tele-assessment were broadly consistent with in-person developmental assessment scores (Ozonoff et al., 2024).

#### Clinical Interview

Clinicians completed a clinical interview with caregivers focused on child social and communication skills, as well as restricted, repetitive patterns of behavior. Clinical interviews were guided by interview procedures used in prior work (Corona et al., 2024; Gangi, 2025).

#### Caregiver Questionnaires

Before the tele-assessment visit, caregivers completed one questionnaire collecting demographic information, medical history, developmental history, and any concerns about tele-assessment. Following tele-assessment, caregivers completed a questionnaire assessing their perceptions of the tele-assessment procedures. Post-assessment questions used Likert-style responses (i.e., “Not true,” “somewhat true,” “very true”) to elicit feedback on caregivers’ understanding of clinician-delivered instructions, comfort during tele-assessment, and their perceptions of the duration and content of the activities. Questionnaires were previously developed for use in assessing family perceptions of tele-assessment and have been used in past published tele-assessment research (Corona et al., 2024; Gangi, 2025).

#### Clinician Questionnaire

Following tele-assessment, clinicians documented observed and reported characteristics of autism according to DSM-5 criteria, their ultimate diagnostic impressions (i.e., Autism/Not Autism; Autism/Not Autism/Unsure), and diagnostic certainty (i.e., “completely certain,” “somewhat certain,” “somewhat uncertain,” or “completely uncertain”). This four-point diagnostic certainty scale has been used in past work (Corona et al., 2024; Gangi et al., 2025; Wagner et al., 2020) and has been associated with diagnostic outcome and clinician satisfaction. Clinicians also provided feedback about tele-assessment procedures, including child, caregiver, or technology factors impacting the assessment. As above, clinician questionnaires were developed for past research on tele-assessment and have been utilized in past published work (Corona et al., 2024; Gangi et al., 2025).

### In-person Assessment Measures

#### Mullen Scales of Early Learning (MSEL)

The MSEL (Mullen, 1995) is a standardized developmental assessment normed for children through age 68 months. It provides scores for Visual Reception, Fine Motor Skills, Receptive Language, and Expressive Language, as well as an overall index of ability, the Early Learning Composite. Clinicians administered either the MSEL or the Differential Abilities Scale, Second Edition (DAS-II), based on clinical judgment and child presentation. The MSEL was administered primarily with children with minimal verbal language and those who demonstrated limited engagement with DAS-II activities. Past research has documented acceptable convergent validity of the MSEL and the original Differential Ability Scales (Bishop et al., 2011).

#### Differential Abilities Scale, Second Edition (DAS-II)

The DAS-II (Elliott, 2007) is a standardized cognitive assessment with norms for children from two years, six months of age through 17 years, 11 months of age. The DAS-II has strong internal reliability (average reliability coefficients range from 0.79 to 0.94) and is often used in clinical research related to autism (Saulnier, 2013). It provides scores for Verbal, Nonverbal Reasoning, and Spatial domains, as well as a Global Conceptual Ability score. As noted above, clinicians administered either the MSEL or DAS-II based on clinical judgment. The DAS-II was administered most often for children communicating with spoken phrases or sentences.

#### Vineland Adaptive Behavior Scales, Third Edition (VABS-3)

The VABS-3 (Sparrow, 2016) is a standardized, clinician-administered caregiver interview that assesses adaptive functioning in social, communication, and daily living skills. Normed, standard scores are provided for birth through adulthood for each domain, as well as an overall Adaptive Behavior Composite. The Vineland-3 has strong interrater and inter-interview reliability (reliability coefficients ranging from 0.70 to 0.81) (Hill et al., 2017).

#### Autism Diagnostic Observation Schedule, Second Edition (ADOS-2)

The ADOS-2 (Lord, 2012; Luyster et al., 2009) is a semi-structured, play-based interaction and observation designed to assess characteristics of autism, with distinct modules designed for use based on a child’s language level and age. The ADOS-2 Calibrated Severity Scores (CSS) have strong test–retest reliability and are considered a stable indicator of the presence of autism-related behaviors across time (Janvier et al., 2022).

#### Caregiver questionnaire

Following in-person assessment, caregivers completed a brief questionnaire providing feedback on in-person assessment procedures. Questions were based on those used following tele-assessment appointments, with language adjusted to reflect in-person procedures. Open-ended questions asked for feedback comparing tele- and in-person assessment.

#### Clinician Questionnaire

Clinicians documented diagnostic impressions, diagnostic certainty, and characteristics of autism, as well as any child or caregiver behaviors impacting the assessment. As above, questions were aligned with tele-assessment questionnaires, with wording adapted to fit the in-person context.

### Procedure

Study procedures were completed in accordance with IRB-approved protocol. All participating families completed informed consent with a research team member. Tele-assessment and in-person assessments were all completed by psychological providers (n = 10) with expertise in assessment and diagnosis of autism in young children, including ADOS-2 research reliability. All psychological providers were involved in the development of the TAP-P.

Following informed consent, caregivers completed a pre-appointment questionnaire, as described above. Participants then completed a tele-assessment appointment with a licensed psychological provider. Tele-assessment appointments included administration of the TAP-P, the DP-4, and a clinical interview focused on autism-related behaviors. Following the tele-assessment, caregivers completed a questionnaire documenting their impressions and feedback on the appointment. Clinicians completed a questionnaire documenting their diagnostic impression, diagnostic certainty, and feedback on the tele-assessment procedures. Diagnostic impressions were not shared with families following tele-assessment, as all families received in-person assessment as part of the study design.

In-person assessments were completed, on average, within two weeks of tele-assessment (m = 12.73 days, SD = 15.91, range 1–98 days). The majority of participants (94%) received in-person assessment within one month of tele-assessment. In-person assessments were administered by a licensed psychological provider unaware of tele-assessment outcomes or impressions. In-person assessment included cognitive (DAS-II) or developmental (MSEL) assessment, adaptive behavior assessment (VABS-3), administration of the ADOS-2, and clinical interview with caregivers focused on autism-related behaviors. Following in-person assessment administration, clinicians shared diagnostic feedback and recommendations with families verbally and prepared a written report.

## Results

Following in-person evaluation, 83% of children (n = 96) across the full sample were diagnosed with autism. Autism was ruled out for 17% of children (n = 20) following in-person evaluation, with clinical best estimate diagnoses reflecting other delays (n = 18), anxiety (n = 1), and typical development (n = 1).

Following tele-assessment, clinicians were asked to document their diagnostic impressions by indicating whether the child’s presentation was consistent with autism (“autism present”), not consistent with autism (“autism absent”), or whether the clinician was unsure. Clinicians indicated “autism present” in 50% of cases, “autism absent” in 10% of cases, and “unsure” in 41% of cases. Additional analyses were completed to investigate factors contributing to diagnostic ascertainment (i.e., the clinician’s ability to arrive at a diagnostic conclusion via tele-assessment). Pearson chisquare analysis indicated that diagnostic ascertainment did not differ for children who did vs. did not identify as from a racial or ethnic minority background (χ = 2.38, *p* > .05) or for children with family income below vs. above the state median income (χ = 2.52, *p* > .05). Diagnostic ascertainment did not differ as a function of assessing clinician (χ = 7.77, *p* > .05). Instances of diagnostic ascertainment and instances of diagnostic uncertainty following tele-assessment were reported by all participating clinicians.

Diagnostic ascertainment did differ as a function of child verbal language level. Tele-assessment clinicians selected “unsure” more often for children with more verbal language (52% of children assessed using TAP-P Form 2) compared to the children with less verbal language (28% of children assessed using TAP-P Form 1), reflecting a statistically significant difference (Pearson chi-square χ = 6.80, *p* < .05). Given this difference in diagnostic ascertainment, separate analyses were completed for children assessed using TAP-P Form 1 and TAP-P Form 2, which are discussed in turn below.

Clinicians were also asked to document their impressions when given only a choice between “autism present” or “autism absent.” Within the forced choice parameters, tele-assessment clinicians’ impressions were in agreement with in-person diagnostic outcomes for 89% of children with less verbal language (TAP-P Form 1) and for 76% of children with more verbal language (TAP-P Form 2). Rates of diagnostic agreement were not statistically significantly different as a function of the form used (Pearson chi-square χ = 3.33, *p* > .05). For Form 1, diagnostic disagreements within the forced choice paradigm (n = 6) included five cases in which the tele-assessment clinician indicated “autism absent” and the in-person clinician diagnosed autism. In one case, the tele-assessment clinician indicated the presence of autism, and the in-person clinician ruled out diagnosis of autism. For Form 2, diagnostic disagreements within the forced choice paradigm (n = 15) included 12 cases in which the tele-assessment clinician indicated “autism absent” and the in-person clinician diagnosed autism. In three cases, the tele-assessment clinician indicated the presence of autism, and the in-person clinician ruled out diagnosis of autism. Across instances of diagnostic disagreement within the forced choice paradigm, tele-assessment clinicians indicated “unsure” when given the option to do so for 81% of cases.

### TAP-P Form 1

Among children assessed using TAP-P Form 1 (n = 54), 93% were ultimately diagnosed with autism following in-person assessment. Tele-assessment clinicians correctly identified autism for 69% of children, correctly ruled out autism for 2% of children, and indicated “unsure” for 28%. Diagnostic disagreement occurred for one child, with tele-assessment impression reflecting the presence of autism and in-person assessment ruling out autism (See Table 4).

Independent sample t-tests were used to compare tele-assessment scores for children correctly identified as having autism following tele-assessment (n = 37) and children for whom the tele-assessment clinician reported “unsure” n = 15. Children for whom diagnostic disagreement occurred (n = 1) and for whom autism was correctly ruled out following tele-assessment (n = 1) were not included in this analysis, but their scores are included descriptively in Table 5. Children correctly identified as having autism through tele-assessment had statistically significantly higher TAP-P tele-assessment scores (m = 24.11, SD = 3.55, range 17–29) than children in the “unsure” group (m = 17.80, SD = 2.11, range 15–21; *t* (42.67) = 7.90, *p* < .05). Children correctly identified as having autism also had lower standard scores on the Communication (m = 56.32, SD = 18.51) and Social (m = 57.43, SD = 8.38) domains of the DP-4, compared to children for whom tele-assessment clinicians were unable to make a diagnostic determination (Communication m = 69.64, SD = 12.73, *t* (34.21) = 2.92, *p* < .05; Social m = 66.71, SD = 10.30, *t* (49) = 3.31, *p* < .05).

Tele-assessment clinicians were also asked to document their diagnostic certainty on a four-point scale, with 4 indicating “completely certain” and 1 indicating “completely uncertain.” As expected, tele-assessment clinicians’ diagnostic certainty was higher for children correctly identified as having autism (m = 3.68, SD = 0.48) compared to children in the “unsure” group (m = 1.64, SD = 0.84; *t* (16.23) = 8.54, *p* < .05; See Fig. 2).

Following in-person assessment, the majority (87%, n = 13) of children for whom tele-assessment clinicians were unable to make a diagnostic determination ultimately received a diagnosis of autism. Statistical comparison of in-person assessment scores among children assessed using TAP-P Form 1 was not completed due to the small sample of children not diagnosed with autism (n = 4). Descriptively, children who did not receive an autism diagnosis had in-person assessment scores in the low range on the ADOS-2, reflecting few autism characteristics, and adaptive scores in the low to moderately low range on the VABS-3. Children who did receive autism diagnosis had mean ADOS-2 scores in the high range and mean VABS-3 scores in the low range across domains. Mean scores on developmental and cognitive assessment were in the low range for all children assessed using TAP-P Form 1 procedures (See Table 5).

### TAP-P Form 2

Among children assessed using TAP-P Form 2, 74% were ultimately diagnosed with autism following in-person assessment. Tele-assessment clinicians correctly identified autism for 32% of children, correctly ruled out autism for 11% of children, and indicated “unsure” for 52%. Diagnostic disagreement occurred for three children, with tele-assessment impression reflecting the absence of autism and in-person assessment indicating the presence of autism (See Table 4).

One-way analysis of variance (ANOVA) was used to compare tele-assessment scores among children for whom autism was correctly identified following tele-assessment (n = 19), children for whom autism was correctly ruled out following tele-assessment (n = 7), and children for whom the tele-assessment clinician reported “unsure” (n = 32; See Table 6). Children for whom diagnostic disagreement occurred were kept out of this analysis due to small sample size (n = 3), but their data are included descriptively in Table 6.

One-way ANOVA indicated between group differences in TAP-P scores as a function of tele-assessment clinician diagnostic impression (i.e., autism vs. no autism vs. unsure; *F* (2, 55) = 17.43, *p* < .05). Post hoc analyses using the Games Howell statistic indicated that children identified as having autism following tele-assessment had statistically significantly higher TAP-P scores (m = 23.00, SD = 1.89) than children in the “unsure” group (m = 19.00, SD = 3.09) and children for whom autism was ruled out following tele-assessment (m = 17.57, SD = 1.99). Mean scores were not statistically significantly different for children in the “unsure” group and children without autism.

One-way ANOVA revealed no statistically significant group differences on DP-4 Communication or Social standard scores as a function of tele-assessment diagnostic outcomes. As expected, group differences emerged for clinician certainty (*F* (2, 56) = 20.48, *p* < .05). Clinicians reported higher diagnostic certainty for children correctly identified as having autism (m = 3.30, SD = 0.47) compared to both children in the “unsure” group (m = 2.13, SD = 0.75) and children for whom autism was correctly ruled out (m = 2.43, SD = 0.54; *p*s < .05; See Fig. 2). Diagnostic certainty did not differ when deferring vs. ruling out autism.

Following in-person assessment, 72% (n = 23) of children for whom clinicians reported “unsure” following tele-assessment received a diagnosis of autism. Autism was ruled out following in-person assessment for the remaining 28% (n = 9). Independent sample t-tests were used to compare in-person assessment scores for children who did and did not receive a diagnosis of autism. Children diagnosed with autism had higher scores on the ADOS-2 (m = 7.07, SD = 1.59) than children without autism (m = 3.60, SD = 1.89; *t* = 6.16, *p* < .05). No statistically significant differences emerged for cognitive (DAS-II), developmental (MSEL), or adaptive (VABS-3) scores.

### Clinician Feedback

Clinicians rated their overall satisfaction with tele-assessment using a five-point Likert scale (1 = very unsatisfied, 3 = neutral, 5 = very satisfied). Clinician satisfaction was not significantly different across use of Form 1 (m = 3.45, SD = 1.07) and Form 2 (m = 3.61, SD = 1.41; *t* = 0.72, *ns*). Clinician satisfaction did differ significantly as a function of clinician certainty. Clinicians who reported “unsure” diagnostic impressions reported lower satisfaction (*m* = 2.76, SD = 1.16) than clinicians who indicated a diagnostic decision following tele-assessment (*m* = 4.01, SD = 0.99; *t* (86.18) = 6.02, *p* < .05).

### Caregiver Feedback

Following tele-assessment, caregivers (n = 112) shared feedback about tele-assessment via online questionnaire. Four caregivers did not complete surveys. Of those with completed surveys, caregivers broadly reported high levels of satisfaction with tele-assessment procedures (See Table 7). Overall, 98% of caregivers reported that the psychologists’ instructions during TAP-P procedures were easy to follow, 92% reported that the technology was easy to use, and 90% reported that they would recommend participating in this type of evaluation to others. Fewer caregivers reported that the tele-assessment activities allowed clinicians to see behaviors that most concerned caregivers (52% reported “very true”, 46% reported “somewhat true”).

Independent sample t-tests yielded between group differences on two items, when comparing groups assessed using TAP-P Form 1 vs. Form 2 (See Table 7). Caregivers whose children were assessed using Form 1 group had higher average ratings for items stating “Before I started my child’s telehealth visit, I understood what I would be doing” and “The activities got my child to show the behaviors I am concerned about.”

## Discussion

The present study describes outcomes from a clinical trial examining tele-assessment using the TAP-P, an autism tele-assessment tool designed for preschool children, in comparison to traditional in-person evaluation for autism. Within this work, clear differences emerged in diagnostic ascertainment as a function of a child’s verbal language abilities. That is, clinicians were more able to form a confident diagnostic impression via tele-assessment for children with less verbal language (i.e., children using two-word phrases or less) than for children with more verbal language (i.e., those using flexible phrases or sentences of three or more words). This supports the potential utility of tele-assessment for some preschool-aged children, while emphasizing the critical need for future research focused on understanding for whom tele-assessment may work best.

The design of the current study allowed for differentiation between tele-assessment clinicians’ *ability to arrive at* a diagnostic conclusion and the *accuracy* of their diagnostic conclusions. When tele-assessment clinicians did arrive at a diagnostic conclusion, their diagnostic impressions were consistent with in-person diagnoses at a rate similar to other work in toddler samples (Corona et al., 2024; Gangi et al., 2025). The relatively low rate of diagnostic disagreements between telehealth and in-person assessments, alongside tele-assessment clinicians’ willingness to acknowledge diagnostic uncertainty, speaks to the clinical ability to recognize when evaluation beyond tele-assessment is needed. As such, it is essential for tele-assessment care models to allow for expert clinical judgment and have clear plans for follow-up in cases of diagnostic uncertainty, especially when working with more verbal patient populations.

Among children with flexible spoken language, clinicians were unable to arrive at a diagnostic conclusion following tele-assessment for approximately half of cases. This finding is consistent with past work highlighting the challenges and complexity of tele-assessment of verbal children (Wagner et al., 2023). Following in-person assessment, most – but not all – of these children received diagnosis of autism. As expected, ADOS-2 scores diverged sharply for children who did versus did not receive diagnosis of autism. No statistically significant differences were observed on cognitive or adaptive behavior assessment. Additional work is needed to better understand the profiles of verbal children who do and do not have clear diagnostic presentations via tele-assessment. In particular, future work may clarify behaviors that are observable during ADOS-2 administration that are not evident during tele-assessment.

Consistent with other work (Corona et al., 2024; Wagner et al., 2020), tele-assessment clinicians reported highest diagnostic certainty when making a diagnosis of autism and lower levels of certainty when ruling out autism or reporting an unclear diagnostic conclusion. Clinician satisfaction with tele-assessment procedures was also largely dependent on whether they could arrive at diagnostic conclusions, with lower levels of satisfaction in cases of diagnostic deferral. Together, these findings support tele-assessment using the TAP-P for identifying children with clear presentations of autism. However, further research is needed to understand the best approaches for identifying children and families most likely (and least likely) to be a good fit for tele-assessment.

### Limitations and Future Directions

The present study focused on a sample of children referred explicitly for evaluation for possible autism, with autism-related concerns raised by either families or providers within the community. As such, many participating children ultimately received an autism diagnosis. This sample includes fewer children without autism, reducing the ability to draw conclusions about the ability of the TAP-P to discriminate between autism and other diagnoses, especially among children with less verbal language. In order to better understand the performance of the TAP-P to accurately rule out autism diagnosis, future work must include recruitment of children with a wider range of developmental profiles and developmental concerns not specific to autism.

Relatedly, the relatively small sample sizes when separately analyzing groups assessed using TAP-P Form 1 and Form 2 reduced the ability to draw conclusions related to psychometric properties of the TAP-P. Specifically, for Form 1, the large number of children who ultimately received a diagnosis of autism limited our ability to analyze sensitivity and specificity of TAP-P scores. For Form 2, the wide variability of scores precluded clear recommendations around interpreting scores. Further work will seek to establish traditional psychometric properties within a larger sample of children with a range of developmental profiles, including children without specific referral for autism evaluation.

Finally, this work focused specifically on questions of diagnostic ascertainment and accuracy – that is, can clinicians use tele-assessment to arrive at diagnostic conclusions about the presence or absence of autism, and are they accurate in doing so? There is a pressing need for further work investigating the range of factors that may impact the implementation and success of tele-assessment care models, including (1) the impact and role of varied developmental, cognitive, or adaptive behavior assessment outcomes on clinical decision-making, (2) the role of family preferences, knowledge about child development, and beliefs about autism, and (3) sociocultural and technological barriers to tele-assessment care. Future tele-assessment work must also consider contextual and systems-level factors; though this sample was racially, ethnically, and socioeconomically diverse, the location of this work within a single region of the United States limits our ability to generalize findings to other national and global contexts. Further, as described by Harstad et al. (2023), phenotypic profiles may shift across preschool years in a way that affects diagnostic stability, which also makes designing tools that increase access to care for this age group a challenging but important area of ongoing research.

## Conclusion

The TAP-P is one of the first tools developed specifically for autism-focused tele-assessment among preschool-aged children, who often experience disproportionately long wait times for traditional evaluation. This study highlights potential utility of tele-assessment for some children within this age range. In particular, the TAP-P can support the identification of autism among preschool-aged children with limited verbal language. Past work has documented use of tele-assessment tools with older children with minimal verbal language, using tools designed for younger children (McNally Keehn, 2023). The TAP-P offers a tool specifically developed for the preschool age range, with activities and scoring procedures keyed to this population.

This study also continues to highlight the increased complexity of tele-assessment with children with more verbal language. The lower rate of diagnostic ascertainment among this group is reflective of this increasing diagnostic complexity, yet it is clear that clinicians are able to *identify* such complexity via tele-assessment and refer for additional inperson evaluation. The TAP-P may support identification of autism in children with more verbal language with clearer diagnostic presentations. However, further study is needed to understand the profiles of children with more verbal language who *can* be readily identified via tele-assessment versus those best served through in-person evaluation. It is also possible that factors beyond a child’s profile, such as caregiver concern for autism, caregiver understanding of autism, and factors related to the home environment, may also inform the utility of tele-assessment within this group.

Ultimately, as has been explicated in past work (Zwaigenbaum & Warren, 2020; Zwaigenbaum et al., 2021), it is likely that multiple pathways to diagnostic evaluation are needed to best meet the needs of children and families. Not all pathways will be appropriate for all families. Tele-assessment presents one such pathway that has clinical value for many patients. Future work should continue to examine strategies that can route children and families to the pathways that are the most meaningful, preferred, and clinically appropriate for them. Ideally, such “triage” strategies could ensure that families receive evaluations that are the best fit for them, optimizing autism diagnostic services across systems.

## Figures and Tables

**Fig. 1:**
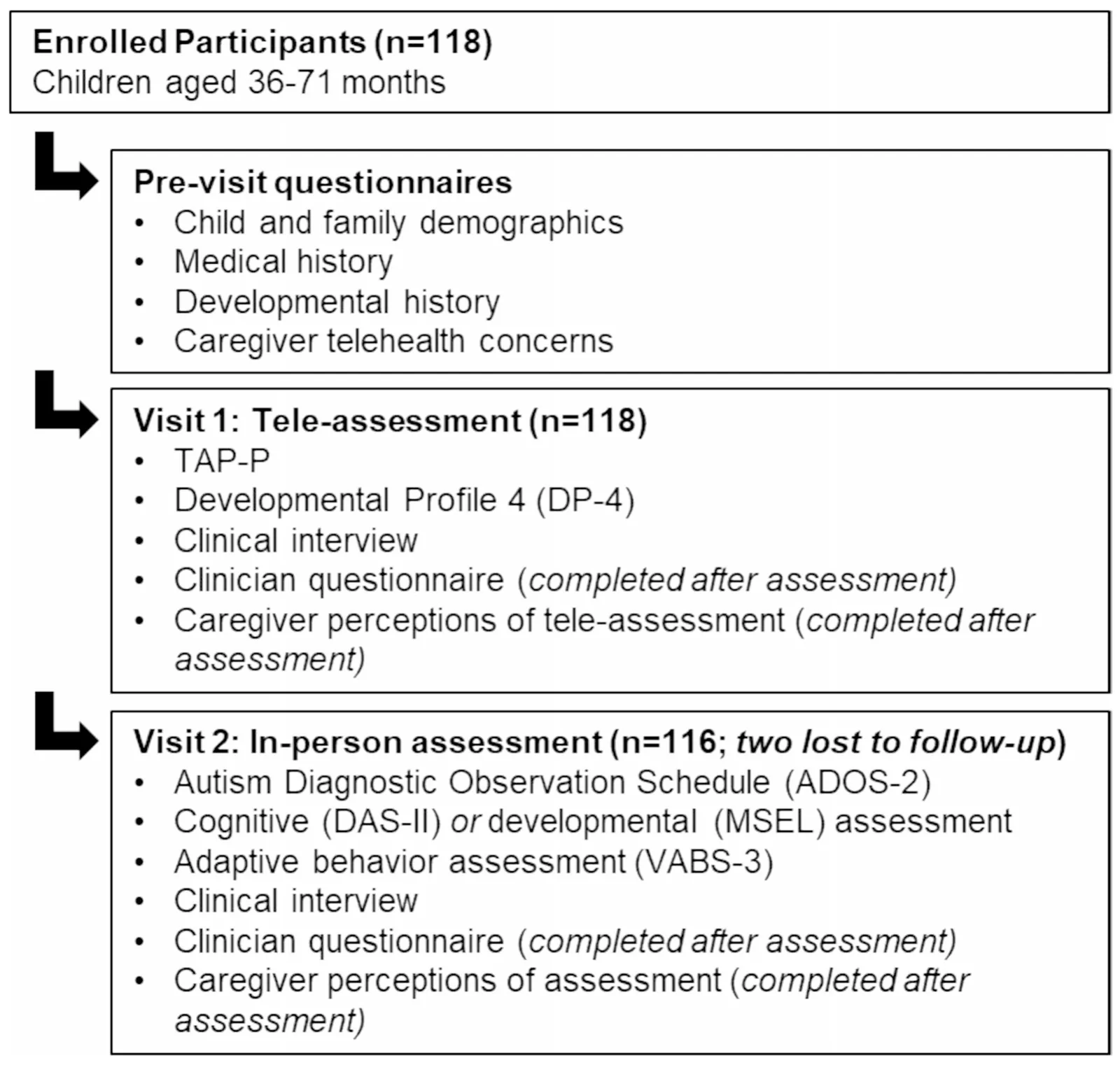
Study flow

**Fig. 2:**
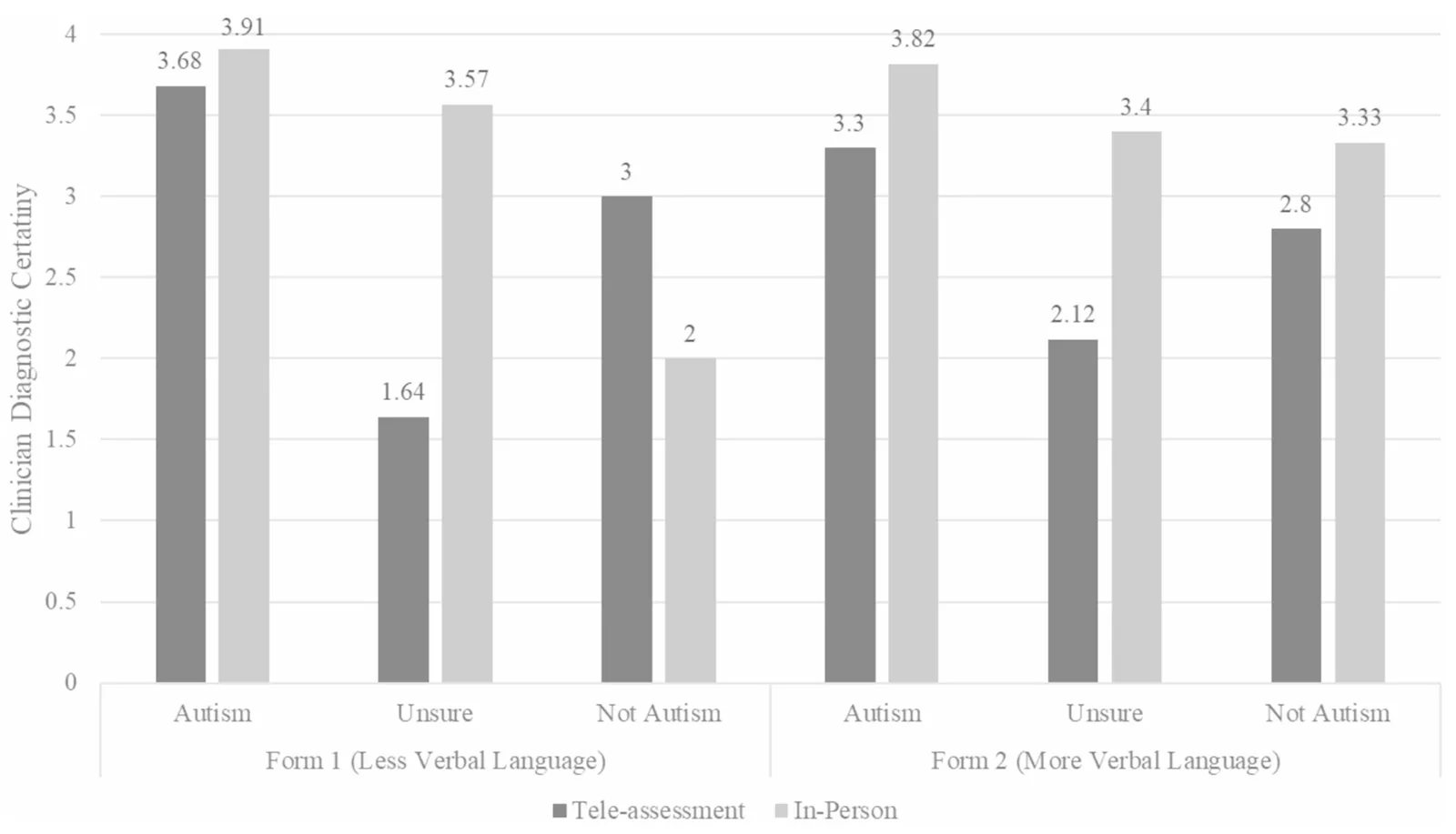
Clinician diagnostic certainty by tele-assessment diagnostic impression

**Table 1:** Participant demographics by TAP-P version administered

	TAP-P Form 1 (Less verbal language) (n = 54)	TAP-P Form 2 (More verbal language) (n = 62)
Child age in months (m [SD])	43.94 [8.60]	51.11 [9.81]
Child sex		
Female	30%	26%
Male	70%	74%
Child race		
Asian	6%	2%
Black or African American	32%	8%
More than one race	11%	15%
White	46%	71%
Opted not to answer	6%	5%
Child ethnicity		
Hispanic or Latino	7%	10%
Not Hispanic or Latino	91%	87%
Opted not to answer	2%	3%
Household Income ($)		
20,000 or less*	11%	18%
20,001–40,000*	30%	7%
40,001–60,000*	9%	21%
60,001–80,000	11%	2%
80,001–100,000	6%	7%
100,001–125,000	9%	7%
125,001–150,000	2%	11%
150,001–200,000	–	3%
Greater than 200,000	6%	13%
Opted not to answer	17%	11%

* Reflects household income below state median income (United States Census Bureau, 2025)

**Table 2:** TAP-P administration activities

Introductory activities (administered to all)	
Independent play	Clinician instructs caregiver to provide 3–5 toys the child usually enjoys; clinician observes independent play
Social responding tasks	Clinician instructs caregiver to call child’s name Clinician instructs caregiver to direct child’s attention to an item across the room by saying “Look!” and pointing
Interactive play	Caregiver is asked to join in with child’s play. Clinician prompts caregiver to initiate pretend play, if none is observed spontaneously
Turn-taking	Caregiver engages in a back-and-forth turn-taking routine (e.g., rolling car or ball, taking turns stacking blocks to build a tower)
TAP-P Form 1 activities (administered to children with less verbal language)	
Interfering	Clinician prompts caregiver to briefly block or interrupt independent play by placing hands on top of a toy the child is using
Social play routine	Caregiver initiates a familiar social routine (e.g., singing a familiar song, playing peekaboo)
Requesting	Clinician prompts caregiver to offer the child two choices (e.g., snacks, preferred toys)
TAP-P Form 2 activities (administered to children with more verbal language)	
Caregiver-led activity	Caregiver is prompted to start a different interactive activity, such as reading a book, singing a song, or talking about a recent family event
Show-and-tell	Clinician shows child an object of interest and tells about it, then prompts child to do the same
Conversation	Clinician prompts conversation with child using questions about child’s preferences and interests
Drawing	Clinician and child each draw a picture, then share what they have drawn with one another
Pictures	Clinician shows the child a series of emotion-laden photos (e.g., a child smiling, child crying) on screen to prompt conversation

**Table 3:** TAP-P items for scoring

Form 1	Form 2
Less verbal language (i.e., two-word phrases or less)	More verbal language (i.e., flexible phrases/sentences of three or more words)

Socially directed speech and sounds	Socially directed speech and sounds
	Speech abnormalities* Conversation*
Quality of vocalizations*	
Frequent and flexible eye contact	Frequent and flexible eye contact
Pointing and other gestures*	
	Use of gestures*
Combines gestures, eye contact, and speech	Combines gestures, eye contact, and speech
	Interactive pretend play*
Repetitive interests/behaviors	Repetitive interests/behaviors
Characteristic or repetitive body movements	Characteristic or repetitive body movements
Sensory exploration or reaction	Sensory exploration or reaction
Quality of social interactions	Quality of social interactions

* Items that differ between Form 1 and Form 2

All items are scored on a three-point Likert scale (3 = behaviors characteristic of autism clearly present; 2 = possible behaviors consistent with autism, and 1 = behaviors characteristic of autism not present), with distinct behavioral anchors provided for each item

**Table 4:** Diagnostic concordance between tele- and in-person assessment outcomes

		Tele-assessment diagnostic impression					

		TAP-P Form 1			TAP-P Form 2		

		(Less verbal language) (n = 54)			(More verbal language) (n = 62)		

		Autism present	Unsure	Autism absent	Autism present	Unsure	Autism absent
In-Person Diagnostic Outcome	Autism present	69%	24%	–	32%	37%	5%
	Autism absent	2%	4%	2%	–	15%	11%

**Table 5:** TAP-P Form 1: Tele- and in-person assessment scores by diagnostic agreement status (telehealth vs. in-person) for children with less verbal language

	ASD-ASD (n = 37)	Unsure-ASD (n = 13)	Unsure-No ASD (n = 2)	ASD-No ASD (n = 1)	No ASD-No ASD (n = 1)
Tele-assessment scores					
TAP-P score (m [SD])	24.11 [3.55]	17.92 [2.22]	17.00 [1.41]	21 [–]	12 [–]
Clinician certainty^*^	3.68 [0.48]	1.67 [0.89]	1.50 [0.71]	3 [–]	3 [–]
DP-4 Social standard score (SS)	57.43 [8.38]	66.17 [11.02]	70.00 [4.24]	64 [–]	84 [–]
DP-4 Communication SS	56.32 [18.51]	67.75 [12.79]	81.00 [2.83]	66 [–]	62 [–]
In-person assessment scores					
MSEL Early learning composite (ELC) [*n* = *39*]^**^	50.14 [2.85]	67.25 [20.13]	49 [–]	49 [–]	65 [–]
DAS-II General conceptual abilitY (GCA) [*n* = 10]^**^	49.25 [18.25]	64.00 [15.81]	74 [–]	–	–
VABS-3 Communication SS	51.28 [16.68]	63.38 [14.31]	70.50 [0.71]	60 [–]	71 [–]
VABS-3 Daily living skills SS	66.56 [9.67]	68.85 [11.73]	77.50 [0.71]	82 [–]	76 [–]
VABS-3 Socialization SS	60.28 [11.04]	64.54 [8.69]	74.50 [6.36]	72 [–]	71 [–]
ADOS-2 CSS	8.37 [1.77]	7.00 [1.22]	3.00 [1.41]	4 [–]	3 [–]

* Clinicians documented diagnostic certainty on a four-point scale, with 4 reflecting completely certain, 3 reflecting somewhat certain, 2 reflecting somewhat uncertain, and 1 reflecting completely uncertain

** Two children were ultimately unable to complete cognitive or developmental testing during their in-person appointment. Three children had partial data (two MSEL; one DAS)

**Table 6:** TAP-P Form 2: Tele- and in-person assessment scores by diagnostic agreement status (telehealth vs. in-person) for children using flexible phrases or sentences

	ASD-ASD (n = 20)^*^	Unsure-ASD (n = 23)	Unsure-No ASD (n = 9)	No ASD-ASD (n = 3)	No ASD-No ASD (n = 7)
Tele-assessment scores					
TAP-P Score (m [SD])	23.00 [1.88]	19.22 [3.40]	18.44 [2.19]	13.33 [1.53]	17.57 [1.99]
Clinician certainty^**^	3.30 [0.47]	2.13 [0.76]	2.11 [0.78]	3.67 [0.58]	2.43 [0.54]
DP-4 Social SS	72.95 [13.78]	75.13 [14.83]	76.44 [22.60]	77.67 [6.43]	75.00 [11.66]
DP-4 Communication SS	86.16 [11.63]	87.09 [18.06]	85.56 [17.48]	89.33 [6.66]	86.29 [13.41]
In-person assessment scores					
MSEL Early learning composite (ELC) [*n* = *7*]	–	69.50 [3.54]	114 [-]	–	71.25 [17.17]
DAS-II General conceptual ability (GCA)[*n* = 48]^***^	90.82 [22.26]	95.56 [19.29]	98.00 [12.23]	91.67 [22.81]	124.5 [9.19]
VABS-3 Communication SS	77.10 (17.75)	80.04 (10.53)	78.78 (15.65)	74.67 (4.04)	82.43 (9.05)
VABS-3 Daily living skills SS	74.55 (10.37)	78.52 (8.31)	73.44 (18.82)	68.33 (5.51)	77.86 (4.98)
VABS-3 Socialization SS	70.65 (11.07)	72.78 (7.56)	75.11 (11.99)	72.00 (7.00)	76.14 (8.78)
ADOS-2 CSS	7.22 (2.37)	7.04 (1.58)	3.11 (1.83)	6.33 (0.58)	4.33 (1.86)

* Complete tele-assessment evaluation data missing for one participant; means for TAP-P and DP-4 scores reflect n = 19

** Clinicians documented diagnostic certainty on a four-point scale, with 4 reflecting completely certain, 3 reflecting somewhat certain, 2 reflecting somewhat uncertain, and 1 reflecting completely uncertain

*** Seven children had partial DAS data (i.e., missing nonverbal or nonverbal and spatial domains; unable to calculate GCA)

**Table 7:** Caregiver perceptions of tele-assessment procedures (m [SD])*

	TAP-P Form 1 (n = 52)	TAP-P Form 2 (n = 60)
Before I started my child’s telehealth visit, I understood what I would be doing**	2.98 [0.14]	2.88 [0.32]
The instructions the psychologist gave me about how to play with my child were easy to follow	2.98 [0.14]	2.98 [0.13]
The activities got my child to show the behaviors I am concerned about**	2.63 [0.53]	2.37 [0.55]
I felt comfortable receiving feedback about my child over telehealth	2.90 [0.36]	2.98 [0.13]
The telehealth visit lasted the right amount of time	2.92 [0.27]	2.90 [0.30]
The telehealth technology was easy to use	2.92 [0.27]	2.92 [0.28]
I would recommend participating in this kind of telehealth evaluation to other caregivers	2.92 [0.27]	2.88 [0.32]

* Caregivers responded to each item using a Likert scale, with 3 = Very true, 2 = Somewhat true, and 1 = Not true

** Independent sample t-tests indicated statistically significant between group differences, *p* < .05
